# Repurposing Canagliflozin to target brain aging

**DOI:** 10.18632/aging.204624

**Published:** 2023-03-24

**Authors:** Hashan Jayarathne, Wanqing Liu, Marianna Sadagurski

**Affiliations:** 1 Department of Biological Sciences, IBio (Integrative Biosciences Center), Wayne State University, Detroit, MI 48202, USA; 2 Department of Pharmaceutical Science, IBio (Integrative Biosciences Center), Wayne State University, Detroit, MI 48202, USA

**Keywords:** sex, Canagliflozin, brain, metabolism, aging

There is a continuous search for pharmacological and nutritional interventions that slow aging. Caloric restriction (CR) is the most intensively studied nutrient intervention that extended lifespan. Anti-diabetic drugs that improve glucose control, reduce postprandial glucose levels, and maintain proper glucose homeostasis can potentially increase lifespan. Sodium-glucose co-transporter 2 inhibitors (SGLT2i) are unique anti-diabetic agents commonly used for the treatment of type-2 diabetes mellitus (T2DM). Their pleiotropic effects include a reduction in cardiovascular disease and all-cause mortality, which are independent of diabetes [1]. Recent studies by the Interventions Testing Program (ITP) have shown that SGLT2i and Canagliflozin extended the median survival of male mice by 14%, and retard age-related lesions in male mice without an effect on females [2, 3].

In the latest study, Jayarathne et al. [4] sought to determine the neuroprotective properties of Canagliflozin during aging. The authors first demonstrated that Canagliflozin treatment improved central insulin sensitivity in aged male mice. Long-term Canagliflozin treatment sensitized the response to insulin by promoting insulin signaling in the hypothalamus and the hippocampus exclusively in aged genetically diverse, UM-HET3 male mice. Canagliflozin treatment further decreased mTOR signaling in a sex-specific manner, predominantly benefiting old males. These findings highlight the protective properties of Canagliflozin treatment beyond those related to systemic glucose control per se. The authors also demonstrated that Canagliflozin treatment significantly reduced neuroinflammation in the hypothalamus and the hippocampus and further improved neuromuscular function, locomotor activity, and anxiety-like behavior in aged male but not female mice [4]. These studies highlight Canagliflozin as a potential neuroprotective agent for the aging brain. However, despite the significance of these observations, the beneficial effects of Canagliflozin were preferentially relevant to male as compared to female animals, indicating sex dimorphism. Mechanistically, Canagliflozin may exert its neuroprotective effects by altering metabolic homeostasis, given its anti-diabetic property. Similarly, another diabetes drug, Acarbose, an intestinal alpha-glucosidase inhibitor, extends the life span mostly in male mice, and improves age-associated hypothalamic neuroinflammation primarily in males but not in female mice [5]. Thus, despite different metabolic modes of action, both anti-diabetic drugs exhibit sex-specific anti-inflammatory effects in the aging brain. Understanding the molecular mechanism underlying this sexual dimorphism could provide insight into the mechanisms critical for neuroprotection.

There could be several reasons for the differences in neuroprotective features between sexes in response to the Canagliflozin treatment including Canagliflozin metabolism and bioavailability in the brain, and sex-specific differences in the *Sglt2* expression levels in different brain regions [4] or different cell populations in the brain. Accordingly, the levels of Canagliflozin detected in the hippocampus differed between sexes when exposed to short or long-term treatment [2, 4] indicating a sex-specific pharmacokinetics. Alternatively, female mice might exhibit some side effects due to the Canagliflozin treatment that oppose the benefits of this drug in the aged brain. Additionally, the sexual dimorphism in brain responses may be linked to the different pathways in the control of aging in either sex. Conversely, SGLT2i actions on the reproductive system may be of importance given that the interactions between sex steroids and neuroinflammation in response to pharmacological intervention in late life were previously documented by our group. Further investigation of these effects would be critical to maximizing the translational potential of Canagliflozin toward drug repurposing for age-associated brain diseases.

SGLT2i increases ketone body concentrations by shifting the fuel consumption from glucose to fat oxidation, resembling CR. It was previously suggested that SGLT2i-mediated protection from diabetes kidney disease involves mTOR inhibition by ketone bodies [6], thus providing the link between SGLT2i inhibition, ketone body metabolism, and nutrient-signaling. In support, Canagliflozin treatment was associated with reduced mTOR signaling and glia activation in the hippocampus of aged male mice [4]. On the other hand, reduced mTOR signaling via rapamycin has been shown to robustly extend lifespan in both sexes in mice. Therefore, the direct or indirect interaction between SGLT2i and mTOR signaling in the brain remains to be clarified. Interestingly, the ability of SGLT2i to reduce inflammatory cytokines in patients with cardiovascular disease was implicated with the inactivation of NLR family pyrin domain containing 3 (NLRP3) inflammasome by SGLT2i via ketone bodies [7]. Similarly, the SGLT2i, Dapagliflozin blocked NLRP3 inflammasome activation via the TCA cycle in chronic kidney disease [8]. Activation of NLRP3 has been linked to brain aging and degenerative diseases. Thus, some of Canagliflozin’s neuroprotective effects may be mediated via the inhibition of inflammatory pathways and the effect on nutrient signaling, which are the two major drivers of aging.

In summary, novel findings demonstrating Canagliflozin neuroprotective effect in the aged brain and its interaction with aging pathways, suggest its future therapeutic potential for age-related brain diseases. If the benefits of the drug are confirmed in longitudinal studies, thus, Canagliflozin should be tested for age-related neurodegenerative diseases to improve health outcomes and quality of life for a large group of society.
